# Combining activity and grimace scores reflects perinatal stability in infants <32 weeks gestational age

**DOI:** 10.1038/s41390-024-03130-6

**Published:** 2024-03-22

**Authors:** Zachary Tuttle, Calum Roberts, Peter Davis, Atul Malhotra, Kenneth Tan, Risha Bhatia, Lindsay Zhou, Elizabeth Baker, Kate Hodgson, Douglas Blank

**Affiliations:** 1 The Ritchie Centre, Monash University, Clayton, VIC Australia; 2 Hudson Institute of Medical Research, Monash University, Clayton, VIC Australia; 3 Women’s Newborn Research Centre, The Royal Women’s Hospital, Parkville, VIC Australia

## Abstract

**Background:**

Over 95% of infants less than 32 weeks gestational age—very preterm infants (VPTI)—require cardiorespiratory support at birth. Clinical condition at birth is assessed by the Apgar score, but the precision and accuracy of activity and grimace has not been evaluated. We hypothesised activity and grimace could predict the level of cardiorespiratory support required for stabilisation.

**Methods:**

Two hundred twenty-nine videos of VPTI resuscitations at Monash Children’s Hospital and The Royal Women’s Hospital, Melbourne were evaluated, with 78 videos eligible for assessment. Activity and grimace were scored (0, 1, or 2) by seven consultant neonatologists, with inter-rater reliability assessed. Activity and grimace were correlated with the maximum level of cardiorespiratory support required for stabilisation.

**Results:**

Kendall’s Coefficient of Concordance (W) showed strong interobserver agreement for activity (*W* = 0.644, *p* < 0.001) and grimace (*W* = 0.722, *p* < 0.001). Neither activity nor grimace independently predicted the level of cardiorespiratory support required. Combining activity and grimace showed non-vigorous infants (combined score <2) received more cardiorespiratory support than vigorous (combined score ≥ 2).

**Conclusion:**

Scoring of activity and grimace was consistent between clinicians. Independently, activity and grimace did not correlate with perinatal stabilisation. Combined scoring showed non-vigorous infants had greater resuscitation requirements.

**Impact:**

- Our study evaluates the precision and accuracy of activity and grimace to predict perinatal stability, which has not been validated in infants <32 weeks gestational age.
- We found strong score agreement between assessors, indicating video review is a practical and precise method for grading of activity and grimace.
- Combined scoring to allow a dichotomous evaluation of infants as non-vigorous or vigorous showed the former group required greater cardiorespiratory support at birth.

## Introduction

The Apgar score is the most widely used method for recording initial newborn status. It may be used to assess perinatal stability and identify infants that require closer observation in the hours after delivery.^1^ The Apgar score consists of five components: appearance (colour), pulse (heart rate), grimace (reflex irritability), activity (tone) and respiration (respiratory effort).^2^ There is currently limited understanding of the interobserver agreement (precision) of scores for initial activity and grimace and whether these scores independently predict resuscitation requirements at birth (accuracy).

Over 95% percent of very preterm infants (VPTI) receive respiratory support at birth, with 12–25% requiring emergency endotracheal intubation in the delivery room.^3,4^ Initial respiratory support influences survival and morbidity.^5^ These outcomes can be improved by avoiding unnecessary mechanical ventilation in stable infants whilst escalating care in those struggling with the transition to independent breathing.^6,7^ Early identification of infants who can achieve cardiopulmonary stability with spontaneous breathing on continuous positive airway pressure (CPAP) may have utility in minimising unnecessary escalations in resuscitation and potential complications.

Video recording offers a high-fidelity tool to assign the components of the Apgar scores.^8^ Review of video recordings of neonatal resuscitation has led to improvements in clinical care.^9–11^ Considering the degree of variability and potential bias in Apgar scoring, the validity of scores allocated at the time of birth is uncertain.^12,13^ Video recording provides the opportunity for multiple observers to focus on assessment without the need to simultaneously provide clinical intervention.^14^

We hypothesised that initial activity and grimace of VPTI assessed using video recordings reliably predicts the level of respiratory support required to achieve cardiopulmonary stability during neonatal transition. We also aimed to compare differences in heart rate, oxygen saturations (SpO_2_), airway pressures and oxygen requirements between groups.

## Methods

This was a retrospective review of data collected from two large perinatal centres—Monash Children’s Hospital (MCH) and The Royal Woman’s Hospital (RWH) in Melbourne, Australia. Each centre averages >7000 annual births and has a NICU with over 58 beds.

Our study received ethics approval with a waiver of parental consent (Monash Health Ref No: RES-22-0000325Q). Eligible videos of neonatal resuscitation of infants were collected at MCH as an ongoing quality assurance project (Monash Health Ref: RES-19-0000-647Q), while videos collected at the RWH were from two clinical trials with parental consent and ethics approval (SHINE Trial Ref No: 18/27, ROSE Study Ref No: N/A).^15,16^

### Eligibility criteria

Videos of infants were included in the study if they were born under 32 weeks gestational age and provided an adequate view of the newborn for at least 5 s between placement on the resuscitation bed after birth and provision of initial respiratory support (except for CPAP). We excluded videos of newborns with a significant congenital abnormality. Videos in which a reliable heart rate and SpO_2_ reading was not available within 3 min of birth were also excluded, to ensure these factors infants could be included for our secondary outcome analysis of perinatal stability.

Clinical care, in particular the commencement and escalation of cardiorespiratory support was in accordance with the Australian Resuscitation Council neonatal guidelines.^17,18^ Videos in which infants were intubated prior to a trial of non-invasive support (i.e. planned immediate intubation prior to birth) were excluded.

### Data collection

**Fig. 1:**
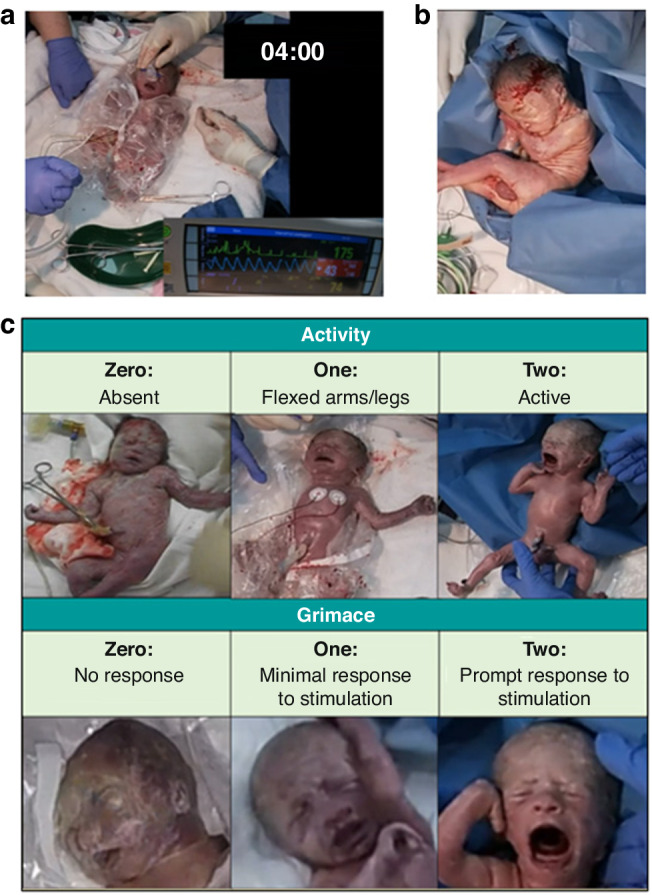
VPTI Resuscitation Video Collection. **a** Screenshot of a resuscitation video of the VPTI and display monitor, used to extract data during neonatal resuscitation. **b** Screenshot of video shown to the blinded assessors of the VPTI on the resuscitation bed. **c** Top: Scoring criteria for Activity; Bottom: Scoring criteria for Grimace.

We reviewed 229 videos of neonatal resuscitations for inclusion (Supplementary Video and Fig. 1a). Videos showed the infant on the resuscitation bed paired with a continuous video of the patient monitor. The alarm of the 1-min Apgar timer was used as a reference point to confirm the time of birth. We manually extracted heart rate and SpO_2_ at 5 s intervals for 5-min after birth from the video of the monitor display, excluding data points with an inadequate QRS or pulse oximetry waveform. Interventions performed within the duration of stabilisation were recorded, including if the team provided CPAP, mask positive pressure ventilation (PPV), or intubation, the peak end expiratory pressure, peak inflation pressure (PIP) and peak fraction of inspired oxygen (FiO_2_). These interventions were measured by direct observation or explicit statement of intention to intervene by the clinical team. Patient characteristics were recorded from medical records.

### Blinded assessment

Seven neonatal consultants assessed video clips without audio of the infant as it was placed on the resuscitation bed (Fig. 1b). The video clips comprised of recordings of the entire resuscitation sequence as the infant was placed on the resuscitation bed. Placement on the resuscitation bed occurred at variable times after birth, with a median time of 55 s (IQR = 36–75). This variation can be attributed to infants receiving either immediate or delayed cord clamping based on local protocol. We subsequently condensed the recordings to short videos (5–20 s) to reflect the attending neonatal team’s initial view of the VPTI, which influences the immediate impression of its clinical condition. Variation in the length of each video clip (5–20 s) shown to assessors was due to individual termination of each video prior to escalation of cardiorespiratory support above CPAP—if required - to ensure blinding to the primary outcome. All infants received stimulation upon placement on the resuscitation bed, upon which their response was used to grade grimace. Assessors were given videos of all eligible infants (*n* = 78) and for each video were asked to provide scores for only activity and grimace 0,1 or 2 based on the criteria shown in Fig. 1c. Assessors were only required to provide their scores once.

Outcomes were then analysed between groups based on the median scores for activity and grimace. We also analysed the combined scores of activity and grimace to create two groups of infants: non-vigorous (combined score < 2) and vigorous (combined score ≥2). Combining scores allowed us to assess the results of a dichotomous evaluation based on initial appearance of the infant.

### Study outcomes

We defined the maximum level of cardiorespiratory support required in the delivery room for stabilisation into three mutually exclusive categories:^19^

- No respiratory support/CPAP only
- PPV defined as provision for longer than 15 s
- Any attempted intubation

Clinical decisions regarding when to escalate cardiorespiratory support for the purpose of resuscitation were consistent across selected infants and in accordance with the Australian Resuscitation Council (ARC).^17^

We also recorded the heart rate and SpO_2_ prior to 5-min after birth to compare bradycardia and hypoxia between groups. The maximum FiO_2_ and airway pressures required within the duration of stabilisation were also recorded. We also compared the assessor’s individual activity and grimace scores with overall Apgar score assigned at birth to assess the level of agreement between the assessors and attending clinical team.

### Statistical analysis

Kendall’s Coefficient of Concordance (W) was used to determine inter-rater variability amongst the assessors.^20^ We assessed variability in the level of cardiorespiratory support received between groups using the Kruskal-Wallis H test for activity and grimace and Mann-Whitney U for combined scores. We compared differences in mean heart rate and SpO_2_ between 3–5 min after birth using a 2-way mixed ANOVA because this was the earliest time that data was consistently available. As data were missing for peak FiO_2_ and airway pressures from the RWH cohort, we did a sub-group analysis using the Kruskal-Wallis H Test for activity and grimace and Mann-Whitney U for combined scores. We used IBM SPSS Statistics Version 28 (New York) for all statistical analysis. Statistical significance was considered at *p* < 0.05.

## Results

### Study patients

**Fig. 2:**
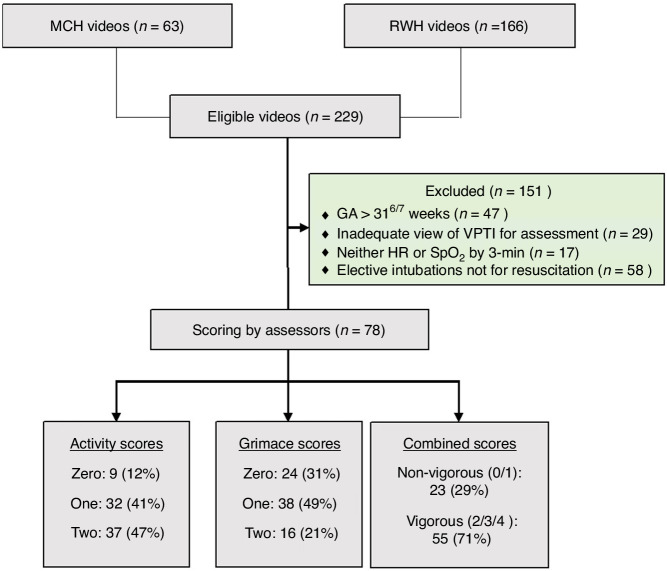
Study Participant Flow Diagram.

There were 229 videos available from the combined databases including, 63 from MCH and 166 from the RWH. Of these videos, 47 videos were excluded as the VPTI’s gestational age exceeded 32 weeks. Twenty-nine videos did not satisfy the criteria to adequately observe and assess the initial activity and grimace prior to initiating ventilatory support above CPAP. Seventeen videos were excluded as neither heart rate nor SpO2 as detected by a reliable QRS waveform was obtained by 3-min. Of the infants intubated, we excluded 58 videos that did not comply with the ARC guidelines for intubation for the goal of resuscitation. After exclusions, 78 videos of individual infants (30 from MCH and 48 from RWH) were scored by assessors blinded to clinical outcomes (Fig. 2).

### Inter-rater reliability

Inter-rater reliability between the seven assessors for activity and grimace was *W* = 0.657, *p* < 0.001 and *W* = 0.722, *p* < 0.001, respectively, demonstrating strong agreement. For activity: 9 infants scored 0 (12%), 32 scored 1 (41%) and 37 scored 2 (47%). For grimace: 24 infants scored 0 (31%), 38 scored 1 (49%) and 16 scored 2 (21%). Using the combined score, 23 infants were classified as non-vigorous—combined score <2 (29%)—and 55 were vigorous—combined score ≥2 (71%). The median length of time of the video clips shown to assessors for review was 15 s (IQR 13–15), with only 9 videos (12%) under 10 s long.

### Patient characteristics

Infants with a grimace score of 0 had a significantly lower median gestational age of 26 (IQR 24–28) weeks compared with those scoring 1 (27, IQR 27–30, *p* = 0.025) and 2 (29, IQR 27–30, *p* = 0.026). No difference was seen in median activity or grimace score based on gestational age below or above 28 weeks. As expected, the median Apgar scores assigned by the attending team were significantly lower for infants scoring 0 compared with those scoring 1 or 2 for activity or grimace and for non-vigorous infants. No significant differences were found for birth weight, sex, delivery mode, type of anaesthesia or antenatal steroids (see Table 1).

**Table 1:** Overall patient demographics and for individual groups (activity and grimace).

		Overall	Activity Score				Grimace Score			
		*n* = 78	0 (*n* = 9)	1 (*n* = 32)	2 (*n* = 37)	*p*	0 (*n* = 24)	1 (*n* = 38)	2 (*n* = 16)	*p*
Gestational Age (weeks)	Median (IQR)	27 (26–29)	28 (24–28)	27 (25–29)	28 (27–30)	0.185	26 (24–28)*	27 (27–30)	29 (27–30)*	0.012
Birth Weight (grams)	Median (IQR)	1011 (735–1287)	1130 (662–1354)	1005 (723–1203)	1040 (811–1280)	0.826	820 (642–1230)	1088 (811–1370)	1097 (935–1260)	0.273
Sex	Male	42 (53.8%)	4 (44%)	21 (66%)	17 (46%)	0.356	15 (63%)	18 (47%)	9 (56%)	0.556
	Female	36 (46.2%)	5 (56%)	11 (34%)	20 (54%)		9 (37%)	20 (53%)	7 (44%)	
Delivery Mode	Vaginal	17 (21.8%)	2 (22%)	6 (19%)	9 (24%)	0.815	5 (21%)	7 (18%)	5 (31%)	0.598
	Instrumental	1 (1.3%)	0 (0%)	0 (0%)	1 (3%)		0 (0%)	1 (3%)	0 (0%)	
	Caesarean Section	60 (76.9%)	7 (78%)	26 (81%)	27 (73%)		19 (79%)	30 (79%)	11 (69%)	
Type of Aeaesthetic^a^	None/Nitrous	19 (24.7%)	2 (22%)	7 (22%)	10 (28%)	0.193	6 (25%)	8 (22%)	5 (31%)	0.283
	Spinal	45 (58.4%)	3 (33%)	19 (59%)	24 (64%)		11 (46%)	24 (65%)	10 (63%)	
	General	13 (16.9%)	4 (45%)	6 (19%)	3 (8%)		7 (29%)	5 (14%)	1 (6%)	
Antenatal Corticosteroids^b^	None	4 (6.9%)	0 (0%)	3 (12%)	1 (4%)	0.450	2 (9%)	2 (7%)	0 (0%)	0.193
	1 Dose	13 (22.4%)	2 (29%)	6 (24%)	5 (19%)		6 (29%)	6 (22%)	1 (10%)	
	≥2 Doses	41 (70.7%)	5 (71%)	16 (64%)	20 (77%)		13 (62%)	19 (71%)	9 (90%)	
1-min Apgar	Median (IQR)	6 (5–8)*	4 (2–5)*	6 (5–7)	7 (6–8)*	<0.001	5 (3–7)*	7 (5–8)	7 (6–8)*	<0.001
5-min Apgar	Median (IQR)	8 (8–9)*	8 (5–8)*	8 (7–9)	9 (8–9)*	0.003	8 (6–8)*	9 (8–9)	9 (8–9)*	0.009

Gestational age rounded down to the nearest week.

^*^ Represents a statistically significant difference between groups (*p* < 0.05).

^a^ Anaesthetics data incomplete (*n* = 1 missing).

^b^ Steroids data incomplete (*n* = 20 missing).

### Primary outcome

Of the 78 infants, 27 (35%) were stabilised with no respiratory support/CPAP alone, 33 (42%) with PPV and 18 (23%) were intubated. No significant difference was found between activity or grimace (0, 1 and 2) for the level of cardiorespiratory support received. However, where the scores were combined, non-vigorous (combined score <2) infants received a greater level of cardiorespiratory support (*U* = 448.5, *p* = 0.03) than vigorous (combined score ≥ 2) infants. The success rate of stabilisation with CPAP alone prior to transfer to the NICU, was lower in non-vigorous infants than vigorous infants (13% vs. 42%, *p* = 0.015).

### Secondary outcomes

**Fig. 3:**
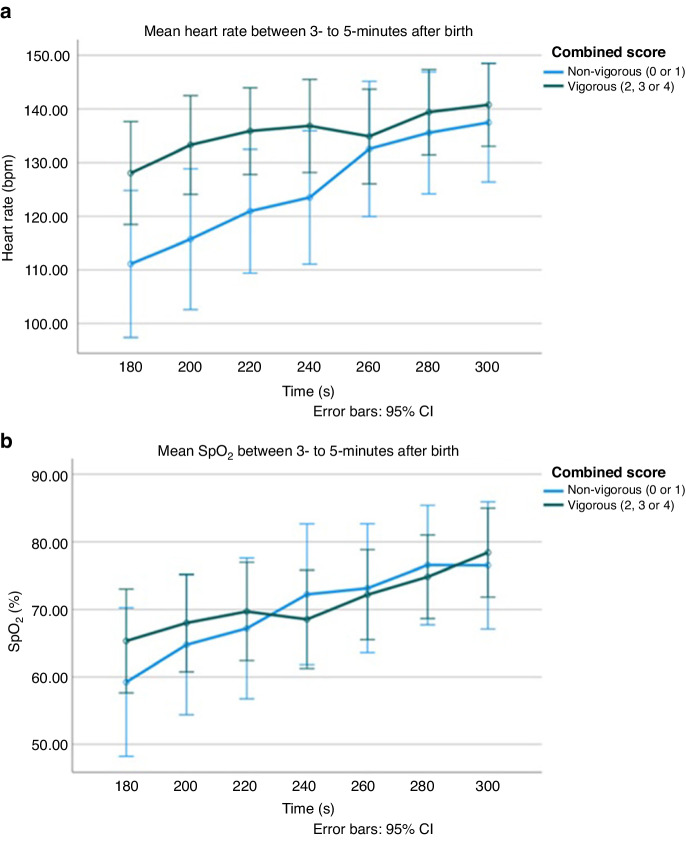
Significant Differences between Non-Vigorous and Vigorous VPTI. **a** Non-vigorous VPTI have a lower 3- to 4-min heart rate than vigorous VPTI, *p* = 0.049 but not between 4- and 5-min. **b** No difference in 3- and 4-min or 4- and 5-min SpO_2_ between vigorous and non-vigorous VPTI.

The individual activity and grimace scores showed poor correlation with the heart rate and SpO_2_ between 3- to 5-min after birth. The non-vigorous infants had a significantly lower mean heart rate between 3- to 4-min but not between 4- and 5-min (Fig. 3a). Mean SpO_2_ between 3- and 5-min did not differ between non-vigorous and vigorous infants (Fig. 3b).

The sub-group analysis of infants with FiO_2_ data available (*n* = 30) showed the median maximum FiO_2_ to be significantly higher in infants with a score of 1 for activity, compared with infants scoring 0 or 2 (*p* = 0.011), activity of 0, FiO_2_ = 40 (IQR 37.5–50), activity of 1, FiO_2_ = 85 (IQR 61.25–100), activity of 2, FiO_2_ = 45 (IQR 30–80). For the same sub-group, the median maximum PIP (*n* = 30) was significantly different for combined scores, with non-vigorous infants having a higher maximum PIP (*p* = 0.049) (non-vigorous, PIP = 30 (IQR 25–30) and vigorous, PIP = 25 (IQR 0–25).

## Discussion

Activity and grimace are important components of the overall Apgar score.^2^ While several studies have investigated the impact of the Apgar score on medium- and long-term neonatal outcomes, our study is the first to assess the relationship between activity and grimace and immediate care after birth.^21–23^ Determining the prognostic value of these factors may assist in guiding appropriate use of resuscitation interventions for VPTI.

We found strong agreement between our assessors for the scoring of activity and grimace. This demonstrates that these components of the Apgar score are consistently applied. All baseline characteristics were similar, except infants with a grimace score of 0 had a significantly lower median gestational age and Apgar scores (1- and 5-min) than those who scored 1 or 2. The correlation with lower gestational age may be due to two factors. The first is that grimace is associated with developmental maturity and diminished in preterm infants. Respiratory effort, muscle tone and grimace have been identified as the major determinants of declining Apgar scores with decreasing gestational age.^24^ Lower Apgar scores in preterm infants have been postulated to reflect depressed neuromuscular responses.^25^ The second theory is that VPTI receive less stimulation and may therefore have longer periods with low Apgar scores.^15^ Infants less than 30 weeks gestational age were observed to receive less stimulation than infants born above (35% vs. 90% respectively) because clinicians provided respiratory support rather than tactile stimulation. Katheria et al. reported that 90% of VPTI breathed spontaneously in the first minute after birth.^26^ Despite stimulation being the first step in neonatal resuscitation, the ideal location, duration and effect of stimulation is poorly defined.^27^ Whilst the role of stimulation in establishing spontaneous breathing is recognised, prospective studies may benefit from the examining the ideal duration and application of stimulation to improve resuscitation outcomes.^28^

The groups based on activity were uneven in number, with sample sizes of *n* = 9 (activity 0), 32 (activity 1) and 37 (activity 2). Infants with a score of 0 for activity are underrepresented, impacting our ability to assess the clinical significance of our findings among activity scores alone. Analysis of the combined score showed non-vigorous infants received more invasive cardiorespiratory support than vigorous infants. We combined scores because a dichotomous evaluation of the infants as vigorous or non-vigorous may be simpler and more easily applicable in guiding immediate resuscitation than using each component separately. There is often overlap between activity and grimace during a rapid assessment of the newborn’s initial respiratory drive. The success rate of non-vigorous infants being stabilised on CPAP was substantially lower than vigorous infants (13% vs. 42%). The maximum airway pressure was also significantly higher in non-vigorous infants. A lower threshold to provide cardiorespiratory support to non-vigorous infants, with prioritisation of tactile stimulation, may improve spontaneous respiratory drive during neonatal resuscitation.

Upon determining the median time of placement on the resuscitation bed to be 55 s after birth, we analysed data between 3- to 5-min. Three min was chosen as the lower threshold to maximise the proportion of subjects with a reliable pulse oximetry trace, which has been reported to take a median of 90 s to obtain upon application.^29^ The non-vigorous infants had a significantly lower mean heart rate with a difference of 15bpm between 3- to 4-min. However, there was no difference in the percentage of vigorous and non-vigorous infants that were bradycardic (<100 bpm) in this period. As increasing heart rate is often the first indication of established lung aeration, a lower mean heart rate suggests non-vigorous infants may face difficulty transitioning from placental to pulmonary gas exchange. A study of infants born above 32 weeks gestational age found infants who received resuscitation had a relatively lower—but not bradycardic - heart rate in the first 3-min than the observational (no-resuscitation) group.^30^ The authors advocated for a more nuanced approach to assessing heart rate than the current dichotomous evaluation of bradycardia as under or above 100 bpm. A mean heart rate difference of 15 bpm in non-vigorous infants may distinguish clinical instability despite not meeting established reference ranges for bradycardia.

While heart rate is widely regarded as the best indicator of clinical condition, this information is often unavailable in the early stages of resuscitation.^10,29^ The assessment of heart rate prior to monitor display relies on palpation or auscultation, both of which are inaccurate and unreliable.^31–34^ While ECG has been shown to display a reliable heart rate faster than pulse oximeter, the latter is used more commonly in neonatal resuscitation.^29^ Katheria et al. reported the median time to attain a reliable heart from pulse oximeter after birth to be ~90 s.^29^ Johnson et al. observed the latency period of detecting heart rate from pulse oximetry was too lengthy to align with the neonatal algorithm guidelines for resuscitation.^35^ The inaccurate or prolonged detection of heart rate can delay the provision of care. Cyanosis is normal after birth and typically seen until SpO_2_ levels reach 80%.^36^ This suggests using cyanosis as a surrogate of clinical condition is potentially unhelpful in the first minute of life. Our study shows that activity and grimace scores alone are unhelpful, but in combination, non-vigorous infants are likely to require higher levels of cardiorespiratory support.

### Limitations

There was a delay in receiving a reliable and consistent reading of heart rate and SpO_2_ in the initial minutes after birth. Consistent with previous studies, the acquisition of early heart rate assessment was problematic due to variation in the timing of ECG/pulse oximeter application and frequency of monitor dropout.^29^ As a result, data collection was inconsistent throughout the first 3-min and incomplete for analysis.

The videos collected for assessment originated from three separate trials. Approximately half of the infants were obtained from the two trials conducted from 2019-onwards, which reflects the conditions of contemporaneous patients who are subject to delayed cord clamping, magnesium sulphate exposure and higher rates of antenatal steroids. In comparison, delayed cord clamping and magnesium sulphate exposure were not standard of care at the time of video collection of the older cohort (2004–2006). However, in the individual patient data meta-analysis performed by Crowthers et al., which compared over 2800 VPTI who were exposed to maternal MgSO_4_ with over 2800 controls, no difference was shown in 5-min Apgar scores and no difference for the need of active resuscitation at birth.^37^ Of the 41 patients from the older cohort, data on antenatal steroid loading was missing for 20 (49%), with 3 known to receive no steroid loading (7%) and 18 receiving at least 1 dose of steroids (44%).

Our assessors had the potential to replay videos in a controlled environment to guide their assessment. Whist this may permit a more accurate assessment of activity and grimace, it does not reflect the clinical impression subconsciously influenced by the Apgar components to guide provisional support during newborn transition. Despite having access to records of the total Apgar scores allocated in the delivery room, we did not have individual component scores to assess the correlation with our assessor’s scores.

Our study was observational and the scoring distribution was uneven. This was particularly prominent among the scores for activity, with a smaller sample for activity of 0 (*n* = 9), compared with 1 (*n* = 32) and 2 (*n* = 37). This may explain the lack of significance for our independent analysis of activity, and the significance gained by combining the scores, which reduced the disparity in group sizes.

## Conclusion

Initial assessment of activity and grimace, allowing characterisation of VPTI as vigorous and non-vigorous, showed the latter group had greater resuscitation requirements at birth. Additionally, infants with lower grimace scores correlated with lower gestational age. Further prospective studies incorporating this assessment may be beneficial to determine how this can be used to better promote spontaneous breathing and avoid unnecessary escalations to invasive ventilation measures.

## Supplementary information

Supplementary Video

## Data Availability

The data that support the findings of this study are openly available in Figshare at https://figshare.com/s/b44e744967b2e55acf79.
